# Nasal deformation by face masks on computed tomography

**DOI:** 10.31744/einstein_journal/2022CE0030

**Published:** 2022-09-19

**Authors:** Rafael Maffei Loureiro, Suheyla Pollyana Pereira Ribeiro, Regina Lucia Elia Gomes, Mauro Miguel Daniel

**Affiliations:** 1 Hospital Israelita Albert Einstein São Paulo SP Brazil Hospital Israelita Albert Einstein, São Paulo, SP, Brazil.

**Figure 1:**
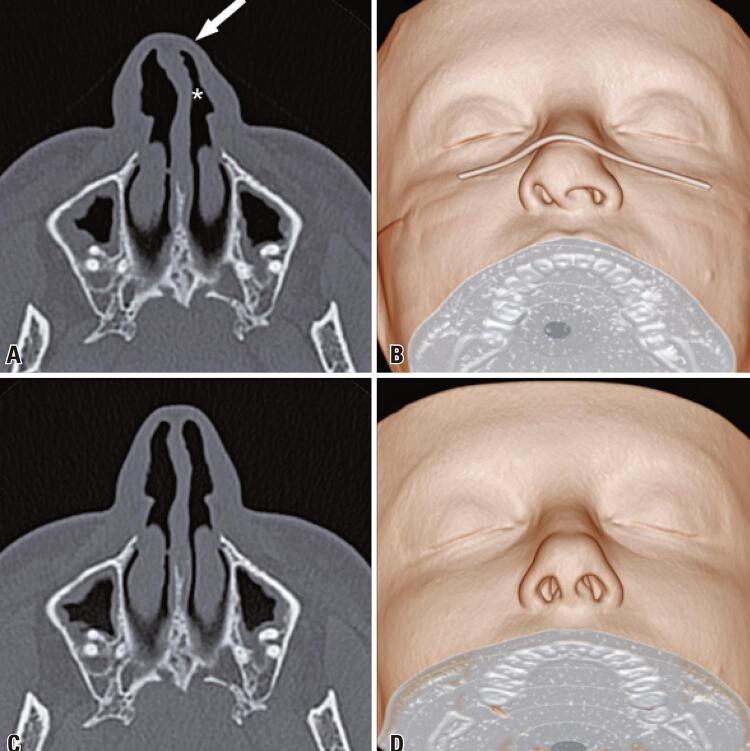
Computed tomography images of the same patient performed in 2021 (A and B) and 2019 (C and D). Axial (A) and three-dimensional volume-rendered (B) computed tomography images show significant deviation of the anterior cartilaginous portion of the nasal septum to the left, with a reduction of the left nasal vestibule (asterisk) and flattening of the nasal dorsum (arrow). These findings are associated with the use of a face mask. Axial (C) and three-dimensional volume-rendered (D) computed tomography images, performed two years before without the use of a face mask, show a slight sinuosity of the cartilaginous portion of the nasal septum; no significant reduction in the nasal air column is observed

Dear Editor,

The widespread use of face masks is an essential tool for slowing the spread of the coronavirus disease 2019 pandemic and its adoption is recommended by the World Health Organization (WHO).^(1)^ During the pandemic, several computed tomography scans of patients wearing face masks showed prominent nasal deformation, such as nasal septal deviations and nasal airway narrowing, which were absent in examinations of the same patients performed before the pandemic (Figure 1). Therefore, care should be taken when evaluating the imaging examinations of the nasal region of patients wearing face masks.

The anterior (cartilaginous) nose plays a key role in the etiology of nasal obstruction since it accounts for 50-75% of the total resistance to airflow in the upper airway.^(2)^ This implies that minor changes in the geometry of the anterior nose may significantly affect nasal function.^(3)^ Similarly, anterior septal deviations tend to cause more airflow resistance than posterior septal deviations.^(4)^

Patients undergoing computed tomography scans for nasal evaluation should be advised to gently loosen their mask during imaging acquisition to avoid nasal deformation and ensure satisfactory imaging interpretation. In addition, since most face masks contain a bendable metal strip that may cause imaging artifacts in the nasal region, a possible recommendation to prevent these artifacts is to provide a metal-free face mask to these patients. In contexts that metal-free face masks are limited, possible solutions would be to invert the mask so that the metal strip is placed under the chin; to remove the metal strip and apply tape across the patient’s nasal bridge; or to remove temporarily the mask just before the imaging acquisition.

Radiologists and clinicians should be aware that wearing face masks during computed tomography examinations might change the patient’s nasal anatomy.
